# Cognitive Control and Flexibility in the Context of Stress and Depressive Symptoms: The Cognitive Control and Flexibility Questionnaire

**DOI:** 10.3389/fpsyg.2018.02219

**Published:** 2018-11-19

**Authors:** Robert L. Gabrys, Nassim Tabri, Hymie Anisman, Kimberly Matheson

**Affiliations:** ^1^Department of Neuroscience, Carleton University, Ottawa, ON, Canada; ^2^Department of Psychology, Carleton University, Ottawa, ON, Canada; ^3^The Royal’s Institute of Mental Health Research, Ottawa, ON, Canada

**Keywords:** stress, cognitive flexibility, cognitive control, emotion regulation, coping, depression

## Abstract

Cognitive control and (cognitive) flexibility play an important role in an individual’s ability to adapt to continuously changing environments. In addition to facilitating goal-directed behaviors, cognitive control and flexibility have been implicated in emotion regulation, and disturbances of these abilities are present in mood and anxiety disorders. In the context of stressful experiences, the reported studies examined processes related to cognitive control and flexibility, emotional regulation and depressive symptoms. To this end, a brief (18-item) self-report measure – the Cognitive Control and Flexibility Questionnaire (CCFQ) – was developed. This questionnaire measures an individual’s perceived ability to exert control over intrusive, unwanted (negative) thoughts and emotions, and their ability to flexibly cope with a stressful situation. In Study 1, the CCFQ was assessed among both university students (*N* = 300) and a community sample (*N* = 302). Preliminary analyses suggested a stable and reliable two-factor structure, that of cognitive control over emotion, and appraisal and coping flexibility. Scores on the CCFQ were strongly associated with greater depressive symptoms, even after controlling for other measures that had been taken to reflect cognitive control and (in)flexibility (e.g., the Ruminative Response Scale; Perseverative Thinking Questionnaire). In Study 2 (*N* = 368), lower scores on the CCFQ were related to more negative stressor appraisals (i.e., greater perceived threat and uncontrollability) of a personally meaningful stressful event. Perceptions of threat and uncontrollability, in turn, partially accounted for the association between CCFQ subscale scores and depressive symptoms. The relation between lower CCFQ scores and heightened depressive symptoms was also partially accounted for by less frequent engagement in problem-focused coping and more use of emotion-focused methods. In Study 3 (*N* = 47 females), lower scores on the cognitive control over emotion component of the CCFQ predicted elevated negative affect and an exacerbated cortisol response following an acute psychosocial stressor (Trier Social Stress Test). The present research points to the CCFQ as a useful self-report tool to identify ways through which cognitive control and flexibility might be manifested in stressful situations, and how reductions in flexibility might be accompanied by elevated symptoms of depression.

## Introduction

Cognitive control and (cognitive) flexibility play a fundamental role in the ability to adapt to continuously changing environments and have been associated with various goal-oriented behaviors, including creativity, problem-solving, multi-tasking, and decision-making (Rolls, 2000; Ionescu, 2012; Dajani and Uddin, 2015). Cognitive control, and the processes underlying this ability (executive functions) have also been implicated in self- and emotional-regulation, as well as mental health outcomes (Gotlib and Joormann, 2010; Hofmann et al., 2012). Impaired cognitive control and flexibility have been observed among depressed individuals and those at risk for the disorder (Murphy et al., 2012; Snyder, 2013; Trivedi and Greer, 2014; Hou et al., 2016). Thus, greater attention has been devoted to identifying factors that contribute to cognitive control and flexibility (Logue and Gould, 2014; Shields et al., 2016), and how disturbances of these abilities might be linked to depressive illness (Davis and Nolen-Hoeksema, 2000; Koster et al., 2011).

Stressful events play an important role in the emergence and maintenance of depressive disorders (Hammen, 2005). Since cognitive dysfunction is often associated with depressive illness, increased efforts have been made at understanding how various types of stressors influence cognitive functioning (Lupien et al., 2009), which may be relevant in identifying potential treatment targets (Koster et al., 2017). Not surprisingly, most studies that have assessed stressor effects on cognitive control and flexibility have done so through neuropsychological or behavioral measures (Liston et al., 2009; Compton et al., 2013; Shields et al., 2016; Goldfarb et al., 2017). These studies have provided insights into how stressors influence fundamental cognitive processes (e.g., working memory, inhibition, set-shifting) and the neurobiological systems mediating these effects (Alexander et al., 2007; Liston et al., 2009; Plessow et al., 2012; Goldfarb et al., 2017). However, such paradigms might provide a narrow perspective of how cognitive control and flexibility might be displayed in stressful experiences, and how reductions of these abilities are manifested in behavioral disturbances. By example, the value of a particular behavioral measure, such as the Wisconsin Card Sorting Task (WCST), might provide an index of flexibility that is limited to certain cognitive domains or situations.

The purpose of the present research was to explore ways in which cognitive control and cognitive flexibility might be expressed in stressful situations that are not readily captured through behavioral tasks. It was also of interest to determine how individual differences in these abilities can serve as resilience or risk factors for depressive pathology. Accordingly, a brief self-report measure – the Cognitive Control and Flexibility Questionnaire (the CCFQ) – was constructed. The CCFQ was modeled after conventional behavioral measures of cognitive control and cognitive flexibility and assesses an individual’s perceived ability to exhibit control over their thoughts and behavior in a stressful situation. Study 1 described the development and psychometric properties of the CCFQ, including the factor structure of this questionnaire and its relationship to other self-report measures which explicitly or implicitly assess processes linked to cognitive control or flexibility. Study 2 tested the hypothesis that greater cognitive control/flexibility (as measured by the CCFQ) would be associated with more favorable stressor appraisals and effective coping, which in turn, would be predictive of lower levels of depressive symptoms. Finally, Study 3 examined whether differences in CCFQ scores moderated the effects of an acute stressor on cognitive, affective, and neuroendocrine responses following the challenge.

### Defining and Measuring Cognitive Control and Cognitive Flexibility

The concepts of cognitive control and of cognitive flexibility have been difficult to define (Morton et al., 2011; Ionescu, 2012; Dajani and Uddin, 2015; Hutchison and Morton, 2016), particularly as their definitions overlap and the same behavioral tasks have been used to assess both abilities (Gläscher et al., 2012; Díaz-Blancat et al., 2018). Generally, cognitive control refers to the ability to focus on information that is currently relevant to a particular goal, while inhibiting information that is not relevant (Morton et al., 2011). Cognitive control is dependent on multiple executive functions, including working memory, inhibition, conflict monitoring, and set-shifting (Gläscher et al., 2012; Mackie et al., 2013), and has often been discussed in terms of facilitating flexible behavioral responses (Miller, 2000; Ridderinkhof et al., 2004; Mackie et al., 2013). Various behavioral tasks have been used to measure specific components of cognitive control, including the n-back task, the Stroop task, the Go/No-Go task, and the WCST, among many others (Botvinick et al., 2001; Harvey et al., 2005; Simmonds et al., 2008; Nyhus and Barceló, 2009).

Cognitive flexibility refers to the ability to modify, or shift between, “cognitive sets” or strategies in response to changes in the environment (Deák, 2003; Stemme et al., 2007; Moore and Malinowski, 2009). This ability has predominantly been assessed by the WCST, the Intra-dimensional/Extra-dimensional (ID/ED) shift task, and the Task Switching paradigm (Grant and Berg, 1948; Downes et al., 1989; Monsell, 2003). Although varying in task specificity, these behavioral paradigms assess the efficiency (or inefficiency) in shifting attention between relevant and irrelevant information. From a cognitive (neuro)science perspective, cognitive flexibility has been viewed as an aspect of cognitive control (i.e., set-shifting) or the manifestation of multiple cognitive control processes which operate sequentially or in parallel (Dajani and Uddin, 2015; Marko and Riečanský, 2018; Zaehringer et al., 2018).

In addition to their characterization as aspects of executive functions, various behaviors are believed to represent cognitive control and flexibility or, at least, be associated with these constructs. For instance, (fluid) intelligence as well as creativity and divergent thinking have been suggested to be expressions of cognitive control and of cognitive flexibility (Colzato et al., 2006; Chermahini and Hommel, 2010; Nijstad et al., 2010; Benedek et al., 2012; Ferguson et al., 2017). Effective problem-solving and decision-making were also proposed to highly depend on cognitive flexibility (Cañas et al., 2003; Isen, 2008; Hare et al., 2009). From a clinical perspective, cognitive flexibility has been described as the ability to change one’s maladaptive attitudes and beliefs with more appropriate ones (Dennis and Vander Wal, 2010), and disturbances of cognitive control were proposed to underpin the use of ineffective emotional regulation strategies, including excessive rumination (Koster et al., 2011). Similarly, in the context of emotional situations, the ability to flexibly attend to and disengage from emotional aspects of a situation or a stimulus, termed ‘affective flexibility,’ might also reflect cognitive control and flexibility processes (Malooly et al., 2013). Although not exhaustive, these examples demonstrate that the terms ‘cognitive control’ and ‘cognitive flexibility’ have clearly been applied to many different behaviors.

Different accounts of cognitive control and cognitive flexibility have led to some confusion as to the defining features of these abilities (Morton et al., 2011; Dajani and Uddin, 2015). Concern has also been expressed that cognitive flexibility lacks a unified definition, and that an unrestricted perspective can result in very different constructs under the same name (Ionescu, 2012). This can lead to theory and research being constructed around mistaken assumptions about what is being measured. On the other hand, a restricted definition of cognitive control or of cognitive flexibility might also prove counterproductive as it can limit the understanding of the role of these abilities in various adaptive and maladaptive behaviors, including the relevance of these constructs to psychopathology. Accordingly, we propose that cognitive control and flexibility can, in fact, be expressed multiple ways. The fundamental factor in the expression of these abilities is highly dependent on *context*, including the characteristics and demands of the situation. For example, it is possible that an individual can be flexible in certain situations (e.g., multitasking) but not in others (e.g., emotional regulation).

### Cognitive Control and Flexibility in the Context Stressful Situations

Cognitive control and flexibility have received a great deal of attention across various contexts and situational (task) demands. However, the ways through which these abilities might be expressed in stressful situations is not fully understood. We propose that cognitive control and flexibility can be manifested through several core processes, which span attention, appraisals/reappraisals, and the endorsement of certain coping strategies.

#### Attention and Attentional Control

Cognitive control can be expressed through directing attention toward information that is relevant to situation (or goal), while inhibiting that which is not relevant. In a stressful situation, this might entail focusing on threat-related information and that which is conducive to eliminating a stressor, and away from non-essential information. As the characteristics of a stressor evolve, this might further comprise shifting attention to newly relevant information, which might represent cognitive flexibility. Stressful events are rarely static or isolated events, and individuals can only attend to a limited amount of information at any given time (Baddeley, 2003). Given the limited capacity of working memory, the ability to focus on relevant information, ignore that which is non-essential, and to flexibly shift attention between multiple sources of information likely serves an adaptive function in coping with continuously evolving stressor experiences.

Stressful events are inherently emotional experiences, and the provocation of negative affective states can influence cognition by biasing information processing toward mood-congruent material (Okon-Singer et al., 2015). Initially, negative emotional responses (e.g., anxiety) might serve an adaptive purpose by directing necessary resources toward distressing stimuli or those requiring coping efforts. However, a preoccupation with negative thoughts and emotions, especially when they are counterproductive in resolving a stressful situation, might limit an individual’s coping effectiveness, and exacerbate the adverse effects of stressors. Extended processing of negative emotional information can result in prolonged and exacerbated negative mood that, over time, might contribute to depressive states (Joormann et al., 2007), and it appears that the inability to disengage from negative emotional information (i.e., cognitive inhibition) might be a defining characteristic of depressive pathology (Gotlib and Joormann, 2010). Likewise, it appears that fundamental cognitive control processes, such as inhibition, play an important role in emotional regulation, where disturbances of cognitive control favor repetitive negative thinking and rumination (Joormann et al., 2007; Koster et al., 2011). Thus, in the context of a stressful situations, cognitive control and flexibility might be expressed through the ability to effectively regulate, or disengage from, negative thoughts and emotions when they no longer serve an adaptive purpose. Diminished cognitive control (and cognitive flexibility), by contrast, might be associated with increased repetitive (or perseverative) negative thinking, rigid information processing (e.g., negative biases in attention and memory), and the maintenance of negative emotional states (Gotlib and Joormann, 2010).

#### Appraisals and Reappraisals

When a potentially stressful situation is first encountered, appraisals are made concerning the extent to which the potential stressor poses a threat and whether the individual has the necessary resources to cope with the experience (Lazarus, 1999; Folkman, 2013). An individual might arrive at an appraisal relatively rapidly, or with little consideration for the context of the situation. Conversely, the appraisal process might occur more slowly and deliberately, whereby the individual approaches the situation from multiple perspectives or contemplates several possible explanations before responding (Lazarus, 1999). Following from this perspective, forming negative appraisals (e.g., interpreting a situation as threatening or uncontrollable) might not necessarily be counterproductive. However, when negative appraisals are applied across situations without considering different characteristics of the various contexts, sustained negative mood is apt to occur (Lackner et al., 2015). Cognitive flexibility might, therefore, involve deliberate appraisal processing, in which multiple alternative appraisals or explanations are determined across stressful situations (Dennis and Vander Wal, 2010).

The appraisal process is dynamic, such that the initial interpretation of a stressful situation, and the perceived ability to cope with it, can be modified, or reappraised, over time (Lazarus, 1999; Folkman, 2013; Cheng et al., 2014). Infrequent engagement in reappraisal processes, or difficulty in doing so, can result in the maintenance of negative affect and has been related to elevated symptoms of depression (Gross and John, 2003; Joormann and Gotlib, 2010). Cognitive control has been suggested to play an important role in the reappraisal process (Ochsner and Gross, 2005; Ochsner et al., 2012), and thus this might be a way through which this ability is expressed in stressful situations. Moreover, the reappraisal process involves shifting “cognitive sets” that are elicited by a stressor, and thus this might be another way through which cognitive flexibility becomes apparent in stressful situations. Consistent with this view, it was suggested that cognitive flexibility allows for shifts between implementation and maintenance of new reappraisals, while working memory is related to the maintenance and monitoring stages of new reappraisals (Zaehringer et al., 2018).

#### Coping Selection and Flexibility

Stressor appraisals are followed by the selection of coping methods to contend with the stressor directly and/or regulate emotional responses (Folkman, 2013). Importantly, although certain coping methods (e.g., problem-solving) seem to be more adaptive than others (e.g., rumination), the effectiveness of any specific strategy or combination of strategies is highly situation-dependent (Matheson and Anisman, 2003). Given this view, optimal coping was proposed to involve flexibility, which can take the form of having a broad and well-balanced coping profile, alternating coping strategies across situations (Cheng et al., 2014). The defining features of coping flexibility closely resemble the hallmark characteristic of cognitive flexibility, which is modifying cognitive or behavioral strategies in response to changing environmental demands. Thus, in the context of a stressful situation, cognitive flexibility might be observed through the ability to generate multiple coping strategies, and to flexibly adjust them according to changing stressor demands. In addition, as mentioned earlier, excessive use of rumination, a coping or emotion regulation strategy characterized by repeated negative thinking concerning one’s dysphoric state (Nolen-Hoeksema et al., 2008), has been hypothesized to be rooted in disturbances of cognitive control (Joormann et al., 2007; Koster et al., 2011). In stressor contexts, cognitive control might, therefore, be accompanied by the increased ability to disengage from repetitive negative thinking (i.e., rumination) which can not only serve to attenuate negative emotional states, but also facilitate proactive coping efforts (Nolen-Hoeksema et al., 2008).

Given that stressful situations are often accompanied by negative emotional responses, cognitive control and flexibility in this context might inherently be tied to the regulation of emotions (e.g., reducing negative emotions or increasing positive emotions through cognitive control). Further, consistent with the conventional view of these constructs (abilities), we consider cognitive control and flexibility to be an individual characteristic that is relatively stable overtime. However, encountering chronic stressors can progressively diminish these abilities (Liston et al., 2009), which could comprise effective emotional regulation. Specifically, chronic stressor exposure can result in continuously engaging limited cognitive resources (e.g., executive functions) required to cope effectively with situational demands. Over time, these cognitive resources may become exhausted, which could be considered a cognitive form of allostatic overload (McEwen, 2003). This, in turn, might lead to greater difficulty engaging in flexible behaviors, resulting in more automatic or reflexive responses and difficulties in regulating emotions.

### Development of the Cognitive Control and Flexibility Questionnaire (CCFQ)

To our knowledge, there are current no self-report measures which directly assess cognitive control in stressful situations. Although it can be argued that the Perseverative Thinking Questionnaire (PTQ; Ehring et al., 2011) and the Ruminative Response Scale (RRS; Treynor et al., 2003) measure products of reduced cognitive control, these questionnaires were not designed to be specific to stressor contexts. The Cognitive Flexibility Inventory (Dennis and Vander Wal, 2010) appears to be the only questionnaire to measure aspects of cognitive flexibility relevant to stressful, or difficult, situations. The developers of this inventory conceptualized cognitive flexibility as being able to challenge and replace maladaptive thoughts with more balanced and adaptive thinking. From this perspective, the Cognitive Flexibility Inventory was designed to measure the tendency to perceive difficult situations as controllable (*control subscale*), the ability to perceive multiple alternative explanations for life occurrences and human behavior, and the ability to generate multiple alternative solutions to difficult situations (*alternatives subscale*).

This inventory measures several important features of cognitive flexibility that might be relevant to depressive disorders. However, it was suggested that the *control* subscale might measure self-efficacy rather than cognitive flexibility (Johnco et al., 2014). Moreover, although the *alternatives* subscale more closely resembles the construct of cognitive flexibility, it was not predictive of cognitive restructuring outcome measures (Johnco et al., 2014). Being a relatively brief (20-item) questionnaire, it understandably does not capture all instances of cognitive flexibility (or of cognitive control) that may be important to stressful situations. Thus, further exploration into how cognitive control and flexibility might be apparent in these contexts might be important, especially in understanding how reductions in these abilities might favor mental health disturbances.

The main purpose of the present research was to develop and evaluate the utility of a novel self-report measure of cognitive control and flexibility that would be specific to stressful situations (the CCFQ). The CCFQ was conceptualized and modeled after the type of cognitive control and cognitive flexibility measured through ‘cold’ executive function tasks (e.g., the Go/No-Go task, the WCST), where the defining features of these measures (inhibiting a prepotent response, shifting cognitive sets or behavioral strategies) served as a foundation for the development of this measure. In order to make these concepts relevant to a stressful situation, cognitive content (i.e., the information that is being manipulated) was operationalized as thoughts, emotions, stressor appraisals, or coping strategies. Control and flexibility were operationalized through statements reflecting shifting, inhibition, updating, including “shifting my attention,” “ignoring,” “setting aside,” “generating multiple …”, “thinking of several ways …”, “reframing,” and “re-evaluating.”

Since stressful experiences often elicit negative emotions, emphasis was placed on how cognitive control and flexibility might be manifested in emotional regulation. From this perspective, the current conceptualization of cognitive control and flexibility can be said to tap into ‘hot’ executive functions. However, being a self-report measure, the CCFQ was not designed to assess ‘hot’ executive functions *per se*, but instead focused on an individual’s perceived levels of cognitive functioning and the implications for emotional regulation and coping effectiveness. Thus, the type of cognitive control and flexibility measured by the CCFQ was intended to converge on the evidence implicating the involvement of basic cognitive control processes in emotion regulation (Koster et al., 2011). Accordingly, the CCFQ was specifically designed to assess an individual’s perceived ability to exert control over intrusive, unwanted (negative) thoughts and emotions, and their ability to flexibly adapt to stressful situations. As previously described, these features of cognitive control and flexibility were proposed to be expressed through three core processes: attention and attentional control, appraisals and reappraisals, and coping selection and flexibility. Finally, the CCFQ was intended to measure trait-like abilities which can, however, be diminished following chronic or prolonged stressor exposure.

## Study 1. Development and Psychometric Properties of the Cognitive Control and Flexibility Questionnaire (CCFQ)

The present study describes the development of the CCFQ as well as the factor structure and reliability of this measure in both a student and a community sample. To evaluate construct validity, a comparison was made between the final (18-item) version of the CCFQ and the Cognitive Flexibility Inventory (Dennis and Vander Wal, 2010). In addition to the Cognitive Flexibility Inventory, we examined the relation between the CCFQ and several previously developed questionnaires that we believed captured features of cognitive control and flexibility (e.g., the Coping Flexibility Questionnaire; Vriezekolk et al., 2012) or reductions of these abilities (e.g., the Perseverative Thinking Questionnaire; Ehring et al., 2011). These additional validation measures were also used to investigate distinct and overlapping features of the Cognitive Flexibility Inventory and the CCFQ. It was hypothesized that the CCFQ would more directly tap into cognitive control and emotional regulation, whereas the Cognitive Flexibility Inventory would be more aligned with flexibility in coping. As a final objective, the present study determined whether the CCFQ was able to capture aspects of cognitive control/flexibility that might be relevant to depressive disorders that are not measured by other questionnaires related to this construct.

### Materials and Methods

#### Item Generation for the CCFQ

A comprehensive review was initially conducted to explore the ways in which cognitive control and flexibility have previously been conceptualized (e.g., executive functions) and assessed (e.g., WCST). A parallel review was undertaken to investigate how control/flexibility might be manifested in stressful situations, which included literature pertaining to attention (set-shifting) and cognitive control as well as stressor appraisals and coping processes. Based on these reviews, 116 items were generated that were believed to reflect cognitive control/flexibility (or reductions of these abilities) in response to stressful experiences, ranging from basic attentional processes to emotional regulation. This initial pool of items was presented to 25 graduate students in psychology and neuroscience who responded to each item and provided feedback as to which of the items were redundant or worded in a confusing or ambiguous manner. Items were further eliminated based on low inter-item correlations (*r* < 0.20), redundancy (*r* > 0.80), confusing or ambiguous wording, or respondents’ interpretations of the item did not fully represent the current conceptualization of cognitive control or of cognitive flexibility. Based on these procedures, 44 preliminary items were retained and used for further evaluation in the student sample in Study 1.

#### Student Sample

##### Participants

Participants comprised 300 undergraduates (*n* = 216 females, 84 males), with a mean age of 19.23 (*SD* = 1.71 years). Self-reported ethnic background included Caucasian (72.6%, *n* = 218), Black (6.0%, *n* = 18), Asian (4.7%, *n* = 14), Arab (4.0%, *n* = 12), South Asian (4.0%, *n* = 12), Hispanic (2.3%, *n* = 7), Aboriginal (1.7%, *n* = 5), South East Asian, 1.0% (*n* = 3), and other (e.g., mixed ethnicity, 3.7%, *n* = 11).

##### Procedure

Participants were recruited through the university’s computerized recruitment system and completed an online survey in which they responded to the 44-item version CCFQ. The CCFQ was introduced as follows:

“The purpose of this questionnaire is to determine what you generally think/feel/do when stressful situations provoke negative thoughts and emotions. Of course, you may act differently depending on the situation, but try to think of what you usually think/feel/do when you are stressed or upset. Using the scale below, indicate the extent to which agree or disagree with the following statements.

Generally, in stressful situations … (specific question follows here)”

Participants responded to each statement on a scale ranging from 1 (strongly disagree) to 7 (strongly agree). In addition to the preliminary version of the CCFQ, participants completed several validation measures, comprising the Cognitive Flexibility Inventory, Coping Flexibility Questionnaire, Emotion Regulation Questionnaire, Ruminative Response Scale, and Perseverative Thinking Questionnaire and their relations to current depressive symptoms. Upon completion of the study, participants received written debriefing and were compensated with course credit.

##### Measures

The *Cognitive Flexibility Inventory* (CFI; Dennis and Vander Wal, 2010) is a 20-item measure that assesses two aspects of cognitive flexibility: (1) the ability to perceive multiple alternative explanations for life occurrences and to generate multiple alternative solutions to difficult situations (*alternatives*), (2) and the tendency to perceive difficult situations as controllable (*control*). Each statement was rated on a scale ranging from 1 (strongly disagree) to 7 (strongly agree). Scores for each subscale were computed by first reversing item scores where relevant, and then summing the appropriate items for each subscale. The internal reliabilities for the *alternatives* and *control* subscales were 0.91 and 0.83, respectively.

The *Coping Flexibility Questionnaire* (COFLEX; Vriezekolk et al., 2012) is a 13-item questionnaire that assesses the capability of switching between assimilative and accommodative coping strategies (*versatility*) and the capability of generating and considering coping options, and appraising the suitability of a coping strategy in a given situation (*reflective coping*). Each statement was rated from 1 (seldom or never) to 4 (almost always), and higher scores indicated greater coping flexibility. Scores for each subscale were computed by summing all relevant items. The internal reliabilities for the *versatility* and *reflective* coping subscales were 0.80 and 0.56, respectively.

The *Emotion Regulation Questionnaire* (ERQ; Gross and John, 2003) is a 10-item questionnaire assessing individual differences in the habitual use of two emotion regulation strategies: *cognitive reappraisal* and *expressive suppression*. All items are rated on a scale from 1 (strongly disagree) to 7 (strongly Agree). Scores were computed by summing ratings for all respective items for the cognitive reappraisal (α = 0.88) and expressive suppression (α = 0.75) subscales, with higher scores indicating greater use of that particular emotion regulation strategy.

The *Ruminative Response Scale* (RRS; Treynor et al., 2003) is a widely used 22-item questionnaire assessing ruminative response styles to sad or depressed mood. For each statement, participants respond on a scale from 1 (almost never) to 4 (almost always) and scores are computed by summing all relevant items. The present study focused on the brooding (α = 0.81) and reflective pondering (α = 0.82) subscales.

The *Perseverative Thinking Questionnaire* (PTQ; Ehring et al., 2011) is a 15-item questionnaire assessing content-independent repetitive negative thinking. Participants responded to each item on a scale 0 (never) to 4 (almost always), and a total score was computed by summing all 15 items (α = 0.95).

The *Beck Depression Inventory* (BDI; Beck et al., 1961) is a widely used 21-item measure that assesses the intensity of depressive symptoms. For each item, participants respond to one of four options which range from low to high depression symptomatology. Total scores were calculated by summing across all 21 items (α = 0.92).

#### Community Sample

##### Participants

Participants in the community sample comprised 302 (*n* = 253 females, 49 males) individuals living in Canada with a mean age of 32.83 (*SD* = 10.36 years). Self-reported ethnic identities included Caucasian (72.8%, *n* = 220), Asian (11.9%, *n* = 36), South Asian (5.0%, *n* = 15), Black (2.0%, *n* = 6), Arab (2.0%, *n* = 6), Aboriginal (1.3%, *n* = 4), Hispanic (1.0%, *n* = 3), South East Asian, (1.0%, *n* = 3), and other (e.g., mixed ethnicity, 2.6%, *n* = 8). Self-reported levels of education included 8 years or less of elementary school (0.3%, *n* = 1), some high school but no diploma (4.3%, *n* = 13), a high school diploma or equivalent (18.9%, *n* = 57), 1 to 3 years of college/university (39.7%, *n* = 120), an undergraduate degree (25.5%, *n* = 77), a master’s degree (7.9%, *n* = 24), a doctoral degree (0.3%, *n* = 1), and a professional degree (e.g., medicine, dentistry) (2.6%, *n* = 8).

##### Procedure

Participants were recruited to participate in a survey called “Coping with Stress” using websites, such as Facebook, Kijiji and Craig’s List, and through word of mouth. In the online consent form, participants were informed that validity checks would be performed on all data to ensure the integrity of responses, and that only those who responded faithfully would receive compensation ($5 gift card to Starbucks or Tim Hortons). Validity checks included (i) the length of time required to complete the survey, (ii) answering 8 out of 12 preselected questions in a non-random way, and completing over half the survey (approximately 70%). Additionally, IP addresses were checked to ensure that the same participant did not complete the survey multiple times. Once consent had been granted, participants completed the shortened (18-item) CCFQ in addition to several related questionnaires. Following the completion of the survey, participants received written debriefing and were mailed their gift card.

##### Statistical analyses

Statistical analyses were performed using SPSS version 20 for Windows (SPSS Science, Chicago, IL, United States). Principal components analyses (PCA) with Promax rotation were used to explore the factor structure of the CCFQ in a student and community sample. Pearson’s correlation coefficients were used to examine the relation between components of the CCFQ, validation measures, and depressive symptoms. Partial correlations determined the strength of relationship between components of the CCFQ and validation measures, after controlling the Cognitive Flexibility Inventory. Partial correlations were also used to assess the strength of relation between components of the CCFQ and depressive symptoms, after controlling each validation measure. As many correlations were generated in the present study, to account for the probability of a Type 1 error, the threshold for statistical significance was adjusted to *p* = 0.0009 (*p* = 0.05/58)

### Results and Discussion

#### Exploratory Factor Analysis: Student Sample

A PCA with Promax rotation (since the factors were expected to be correlated) was conducted to explore the factor structure of the preliminary 44-item CCFQ and to select the final items for this measure. The Kaiser measure of sampling adequacy was 0.95 and Bartlett’s Test of Sphericity was χ^2^(946) = 7724.90, *p* < 0.001. Although inspection of eigenvalues and scree plot for the 44 items suggested a 7-factor solution, only the first 2 factors explained greater than 10% of the total variance across items (Factor 1 = 38.11% and Factor 2 = 11.23%). Furthermore, examination of the unrotated loadings suggested that factors 3 to 7 were not major components as they had fewer than 3 items with substantial (>0.40) factor loadings (see Supplementary Table 1). A parallel analysis (Zwick and Velicer, 1986), in which the actual eigenvalues were compared to average eigenvalues derived from a series of randomly generated data sets (in this case 5000 samples), further supported the presence of two factors.

Based on these analyses, 26 items were eliminated from the initial 44-item pool due to low factor loadings (<0.40) on the two primary factors, were redundant, or were not consistent with the constructs represented by the two primary factors. A second PCA was conducted on the remaining 18 items, in which the number of factors extracted was restricted to two. The Kaiser measure of sampling adequacy was 0.92 and Bartlett’s Test of Sphericity was χ^2^(153) = 2704.62, *p* < 0.001. Table 1 (Student sample) presents the factor structure of the 18-item CCFQ as well as the eigenvalues and percentage of variance accounted for by each of the two factors. Scores for each factor/component were computed by, first, reverse scoring the appropriate items (indicated by the asterisk in Table 1), and then taking the mean of all relevant items (indicated in bold in Table 1).

**Table 1:** Promax-rotated, Principal Components Analysis of the CCFQ in a student and community sample.

	Student sample		Community sample	
		
Item	Cognitive control over emotion	Appraisal and coping flexibility	Cognitive control over emotion	Appraisal and coping flexibility
(1) I get easily distracted by upsetting thoughts or feelings.^∗^ (11)	**0.89**	-0.14	**0.82**	0.07
(2) My thoughts and emotions interfere with my ability to concentrate.^∗^ (16)	**0.85**	-0.16	**0.92**	-0.13
(3) I have a hard time managing my emotions.^∗^ (15)	**0.81**	0.01	**0.82**	0.03
(4) It’s hard for me to shift my attention away from negative thoughts or feelings.^∗^ (18)	**0.81**	-0.02	**0.83**	0.02
(5) I feel like I lose control over my thoughts and emotions.^∗^ (2)	**0.74**	0.11	**0.82**	0.02
(6) It is easy for me to ignore distracting thoughts. (8)	**0.72**	-0.05	**0.45**	0.23
(7) It’s difficult to let go of intrusive thoughts or emotions.^∗^ (4)	**0.70**	-0.09	**0.80**	-0.17
(8) I find it easy to set-aside unpleasant thoughts or emotions. (7)	**0.66**	0.11	**0.33**	0.30
(9) I can remain in control of my thoughts and emotions. (14)	**0.59**	0.19	**0.36**	0.48
(10) I take the time to think of more than one way to resolve the problem. (12)	-0.13	**0.89**	0.02	**0.87**
(11) I approach the situation from multiple angles. (3)	-0.13	**0.88**	-0.05	**0.92**
(12) I consider the situation for multiple viewpoints before responding. (5)	-0.13	**0.86**	-0.23	**0.91**
(13) I take the time to see things from different perspectives before reacting. (10)	-0.10	**0.76**	0.82	**0.82**
(14) I take the time to think of several ways to best cope with the situation before acting. (6)	0.14	**0.68**	0.02	**0.86**
(15) I weigh out my options before choosing how to take action. (1)	-0.02	**0.68**	-0.00	**0.66**
(16) I manage my thoughts or feelings by reframing the situation. (17)	0.26	**0.61**	0.16	**0.74**
(17) I control my thoughts and feelings by putting the situation into context. (13)	0.29	**0.48**	0.13	**0.59**
(18) I can easily think of multiple coping options before deciding how to respond. (9)	0.37	**0.46**	0.10	**0.77**
Eigen value	7.48	2.73	2.37	8.75
Variance Explained (%)	41.57	15.19	13.16	48.62
*Mean* (*SD*)	3.71(1.16)	4.62(0.94)	3.89(1.28)	4.50(1.18)
Cronbach’s α	0.90	0.89	0.90	0.93


*^∗^Indicates reversed items. Bold indicates the factor in which the items were retained. Numbers in parentheses indicate the order in which the items should be administered.*

After examining the items within each factor, Factor 1 was labeled *cognitive control over emotion* and Factor 2 *appraisal and coping flexibility*, The *cognitive control over emotion* dimension comprised items assessing fundamental cognitive control processes (e.g., attention, inhibition, and set-shifting) which might be essential in regulating negative, and potentially irrelevant, thoughts and emotions elicited by a stressful situation. In contrast, the *appraisal and coping flexibility* component measured more complex processes related to changes of appraisals (e.g., approaching a situation from multiple perspectives, and regulating emotions through reappraisal processes) and the generation of a broad range of coping strategies. As indicated by Cronbach’s alpha coefficients in Table 1, the *cognitive control over emotion* and *appraisal and coping flexibility* dimensions of the CCFQ exhibited excellent internal reliability, and the correlation between the two factors was *r* = 0.49, suggesting a moderate degree of overlap.

#### Exploratory Factor Analysis: Community Sample

A PCA with *Promax* rotation was conducted to determine whether the factor structure of the final (18-item) CCFQ observed in the student sample matched that of a community sample. The Kaiser measure of sampling adequacy was.94 and Bartlett’s Test of Sphericity was χ^2^(153) = 3322.95, *p* < 0.001. For this PCA, we did not place a restriction on the number of factors produced. The factor structure of the CCFQ in the community sample was nearly identical to that observed in the student sample, with the exception of one item. In the community sample, the item ‘I can remain in control of my thoughts and emotions’ loaded more strongly on the *appraisal and coping flexibility* component of the CCFQ. However, as this item more substantively represents cognitive control over emotion and loaded more strongly on this factor in the student sample, we retained this item on the cognitive control over emotion factor. The two subscales exhibited excellent inter-item reliability, and were moderately correlated (*r* = 0.61). Together, these preliminary factor analyses and reliability assessments suggest that the CCFQ exhibits a stable two-factor structure in a student and community sample.

#### Construct, Convergent, and Incremental Validity

As shown in Table 2, both subscales of the CCFQ were significantly correlated with nearly all validation measures of cognitive control and (in)flexibility. The strength of the correlations, however, differed for each component of the CCFQ. The *cognitive control over emotion* component was more closely related to the Perseverative Thinking Questionnaire and the Ruminative Response Scale, whereas as the *appraisal and coping flexibility* dimension exhibited a stronger association with the Coping Flexibility Questionnaire. Both components of the CCFQ were similarly associated with the cognitive reappraisal, but not the expressive suppression subscales of the Emotion Regulation Questionnaire. These findings suggest that the CCFQ captures aspects of cognitive control and of cognitive flexibility that might be relevant to stressful situations.

**Table 2:** Zero-order correlations between the CCFQ and validation measures of cognitive control and cognitive (in)flexibility.

	Control (CFI)	Alternatives (CFI)	Versatility (COFLEX)	Reflective coping (COFLEX)	Cognitive reappraisal (ERQ)	Expressive suppression (ERQ)	Brooding (RRS)	Reflective pondering (RRS)	Perseverative thinking (PTQ)
Cognitive control over emotion	0.56^∗^	0.29^∗^	0.41^∗^	-0.01	0.45^∗^	-0.02	-0.57^∗^	-0.35^∗^	-0.73^∗^
Appraisal and coping flexibility	0.36^∗^	0.66^∗^	0.50^∗^	0.23^∗^	0.46^∗^	-0.06	-0.29^∗^	-0.02	-0.35^∗^
CFI Control	–	–	0.47^∗^	0.06	0.29^∗^	-0.19^∗^	-0.45^∗^	-0.22^∗^	-0.55^∗^
CFI Alternatives	–	–	00.41^∗^	0.35^∗^	0.33^∗^	-0.16	0.26^∗^	0.04	-0.19


*CFI, Cognitive Flexibility Inventory; COFLEX, Coping Flexibility Questionnaire; ERQ, Emotion Regulation Questionnaire; RRS, Ruminative Response Scale; PTQ, Perseverative Thinking Questionnaire; ^∗^*p* < 0.0009.*

As expected, the CCFQ was correlated with the Cognitive Flexibility Inventory, suggesting some degree of content overlap between the two questionnaires. As shown in Table 2, the *appraisal and coping flexibility* component of the CCFQ was most strongly associated with the alternatives facet of the Cognitive Flexibility Inventory. Indeed, it is in the measurement of coping processes that the two measures overlap the most with respect to content, which is most noticeable by their relationship to the versatility in coping subscale of the Coping Flexibility Questionnaire. There is, however, one important difference between these two subscales. Whereas the alternatives facet of the Cognitive Flexibility Inventory more directly measures the tendency to generate multiple explanations for difficult situations, the *appraisal and coping flexibility* dimension of the CCFQ assesses the use of reappraisals aimed at regulating negative thoughts and emotions, and difficulties in doing so. In fact, this likely explains why the *appraisal and coping flexibility* dimension of the CCFQ was more strongly correlated with the reappraisal subscale of the Emotion Regulation Questionnaire.

It is important to point out that the way in which ‘reappraisals’ (or the process of reappraising) are assessed by the Emotion Regulation Questionnaire and the CCFQ differs in a subtle, but notable, way. In the Emotion Regulation Questionnaire, reappraisal largely refers to “*changing what I’m thinking about*” to either reduce negative emotions or enhance positive emotions (Gross and John, 2003). In addition to assessing the use of this form of reappraisal (“I manage my thoughts or feelings by reframing the situation.”), the *appraisal and coping flexibility* subscale CCFQ, measures perspective-taking (e.g., considering a situation from multiple viewpoints) as a form of modifying appraisals associated with a stressful situation.

As displayed in Table 2, the *cognitive control over emotion* component of the CCFQ was more strongly linked to the control subscale of the Cognitive Flexibility Inventory. However, the conceptualization and assessment of control differs substantially between the two measures. Specifically, whereas the control subscale of the Cognitive Flexibility Inventory assesses perceived controllability over difficult situations, the *cognitive control over emotions* component of the CCFQ measures the extent to which an individual can exert control of negative thoughts and emotions. To be sure, the CCFQ displayed a stronger relationship with cognitive reappraisal, brooding, and perseverative thinking, whereas the Cognitive Flexibility Inventory was more closely tied to reflective coping and expressive suppression. In essence, unlike the Cognitive Flexibility Inventory, the CCFQ more directly taps into processes related to cognitive control and emotional regulation, most notably, the ability (or inability) to disengage from negative cognitive and emotional states.

To further identify common and distinct feathers between the two scales, partial correlations between the CCFQ and validation measures, controlling for both subscales of the Cognitive Flexibility Inventory, were examined. As shown in Table 3, the *cognitive control over emotion* component of the CCFQ remained strongly associated with cognitive reappraisal, perseverative thinking, and rumination, and the *appraisal and coping flexibility* component was still highly related to coping versatility. In line with the data presented in Table 2, the CCFQ was no longer linked to reflective coping and expressive suppression (Table 3). Thus, on the whole, the CCFQ appears to capture several key elements of cognitive control, coping, and emotion regulation beyond those assessed by the Cognitive Flexibility Inventory.

**Table 3:** Partial correlations between the CCFQ and validation measures of cognitive control and cognitive (in)flexibility after controlling for the Alternatives and Control subscales of the Cognitive Flexibility Inventory.

	Versatility (COFLEX)	Reflective coping (COFLEX)	Cognitive reappraisal (ERQ)	Expressive suppression (ERQ)	Brooding (RRS)	Reflective pondering (RRS)	Perseverative thinking (PTQ)
Cognitive control over emotion	0.18	-0.10	0.35^∗^	0.11	-0.42^∗^	-0.28^∗^	-0.62^∗^
Appraisal & coping flexibility	0.30^∗^	0.00	0.32^∗^	0.08	-0.15^∗^	0.07	-0.25^∗^


*COFLEX, Coping Flexibility Questionnaire; ERQ, Emotion Regulation Questionnaire; RRS, Ruminative Response Scale; PTQ, Perseverative Thinking Questionnaire; ^∗^*p* < 0.0009.*

An important objective of the present study was to determine whether the CCFQ predicted depressive symptoms beyond that of previously developed self-report measures which, either explicitly or implicitly, assessed processes related to cognitive control and cognitive flexibility. As shown in Table 4, after controlling for each validation measure, both subscales of the CCFQ still predicted depressive symptoms. The only notable decrease in the correlation between the CCFQ and depressive symptoms appeared to occur when controlling for perseverative thinking and rumination. This suggests that the CCFQ taps into repetitive (negative) thinking that often accompanies symptoms of depression. Indeed, this was expected given that diminished cognitive control (and cognitive flexibility) is characterized by perseverative responding. The CCFQ, however, addresses other aspects of cognitive control that are related to depressive pathology. For instance, beyond repetitive negative thinking, disturbances of cognitive control can also be expressed by difficulties preventing negative emotional information from entering working memory and an inability to disengage such emotional material once it has been attended to (Gotlib and Joormann, 2010). In this regard, whereas the Perseverative Thinking Questionnaire focuses specifically on repetitive thought independent of content, the *cognitive control over emotions* component of the CCFQ assesses, more broadly, the ability to exhibit control over (primarily negative) thoughts and emotions. This said, it is important to acknowledge the high correlation between the Perseverative Thinking Questionnaire and the *cognitive control over emotions* component of the CCFQ, which suggests a high degree of overlap between the two measures and potential redundancy. Indeed, this raises the possibility that both measures can be used to assess the same processes and administered in the same or similar contexts.

**Table 4:** Partial correlations between the CCFQ and depressive symptoms after controlling for validation measures of cognitive control and cognitive (in)flexibility.

	Depressive symptoms controlling for									
	
	Zero-order correlation	Control (CFI)	Alternatives (CFI)	Versatility (COFLEX)	Reflective coping (COFLEX)	Cognitive reappraisal (ERQ)	Expressive suppression (ERQ)	Brooding (RRS)	Reflective pondering (RRS)	Perseverative thinking (PTQ)
Cognitive control over emotion	-0.55^∗^	-0.41^∗^	-0.52^∗^	-0.49^∗^	-0.55^∗^	-0.47^∗^	-0.56^∗^	-0.35^∗^	-0.47^∗^	-0.24^∗^
Appraisal and coping flexibility	-0.34^∗^	-0.21^∗^	-0.25^∗^	-0.22^∗^	-0.34^∗^	-0.21^∗^	-0.33^∗^	-0.22^∗^	-0.37^∗^	-0.18


*CFI, Cognitive Flexibility Inventory; COFLEX, Coping Flexibility Questionnaire; ERQ, Emotion Regulation Questionnaire; RRS, Ruminative Response Scale; PTQ, Perseverative Thinking Questionnaire. ^∗^*p* < 0.0009.*

Unlike other questionnaires, the CCFQ measures not only whether an individual engages in behaviors reflecting cognitive control and flexibility, but also perceived difficulties in doing so. In fact, it might be these specific perceptions (i.e., level of difficulty in cognitive control) that might predict depressive symptoms beyond the other measures used in Study 1.

It is important to mention that although the CCFQ was designed to assess relatively stable individual characteristics, we have yet to test the stability of this measure over time. Thus, the lack of test-retest reliability of the CCFQ is an important limitation of the present study. This notwithstanding, the present findings support the notion that the CCFQ might be a useful self-report measure of processes related to cognitive control and flexibility in the context of stressful situations. As indicated by its relationship to the validation measures, the CCFQ appears to tap into multiple ways through which cognitive control and flexibility might be expressed in stressful situations in a single, brief questionnaire. At the same time, this questionnaire provides unique predictive utility for understanding depressive symptoms, beyond that of other measures.

## Study 2. The CCFQ in Relation to Stressor Appraisals and Coping Style

Study 2 examined whether scores on the CCFQ were predictive of stressor appraisals and coping style, and whether these processes mediated the relation between cognitive control/flexibility (assessed through the CCFQ) and depressive symptoms. Since the data in the present study were cross-sectional, mediation analyses were not intended to address causality. Instead, these analyses were used to determine whether stressor appraisals, coping strategies, or both accounted for a significant proportion of the variance in the relation CCFQ subscale scores and depressive symptoms. Prior to these analyses, an Exploratory Structural Equation Model (ESEM) was used to confirm the two-factor structure of the CCFQ and to test alternative models.

### Materials and Methods

#### Participants and Procedure

Participants comprised 368 undergraduate students (*n* = 288, 78.3% females, 80, 21.7% males), with a mean age of 19.53 (*SD* = 1.59 years). Self-reported ethnicity included Caucasian (70.9%, *n* = 261), Asian (6.8%, *n* = 25), Black (6.0%, *n* = 22), Arab (5.2%, *n* = 19), South Asian (4.1%, *n* = 15), Hispanic (2.2%, *n* = 8), South East Asian (1.6%, *n* = 6), Aboriginal (0.3%, *n* = 1), and other (e.g., mixed ethnicity, 3.0%, *n* = 11). As in Study 1, participants were recruited through Carleton University’s online recruitment system.

After providing written informed consent, participants reflected on a recent, personally meaningful academic situation that they found stressful; the central theme for most students was “overwhelmed with school work.” They then completed questionnaires assessing stressor appraisals, coping, symptoms of depression, and the CCFQ. Upon completion of the study, participants received written debriefing and were compensated with course credit.

#### Measures

##### Stressor appraisals

The Stress Appraisal Measure (SAM; Peacock and Wong, 1990) assessed several appraisal dimensions in response to the academic stressor including, perceptions of threat, challenge, centrality, control-by-self, control-by-others, and uncontrollable-by-anyone. Ratings were on a scale ranging from 1 (not at all) to 5 (a great amount). Scale scores for each appraisal dimension were calculated by obtaining the mean rating for items comprising each scale. The internal reliability of each appraisal dimension was: threat (*α* = 0.71), challenge (*α* = 0.65), centrality (*α* = 0.84), control-by-self (*α* = 0.85), control-by-others (*α* = 0.89), and uncontrollable-by-anyone (*α* = 0.71).

##### Coping style

Coping style was assessed using the Survey of Coping Profile Endorsement (SCOPE; Matheson and Anisman, 2003). The SCOPE is a 50-item measure assessing the frequency of endorsement of 13 coping strategies. For each item, respondents indicated the extent to which they had demonstrated each of the behaviors as a way of dealing with stressors in recent weeks on a scale of 0 (Never) to 4 (Frequently). Based on previous use of the SCOPE (Matheson and Anisman, 2003) and a principal component analysis, three broad clusters of coping were examined: Problem-focused coping, comprising problem-solving, cognitive restructuring, active distraction, humor, and social support seeking (*α* = 0.85); emotion-focused coping, including rumination, emotional expression, emotional containment, other-blame, and self-blame (*α* = 0.88); and avoidant coping comprising avoidance, wishful thinking, and passive resignation (*α* = 0.74).

##### Depressive symptoms

The 21-item BDI (Beck et al., 1961) was again used to assess the intensity of depressive symptoms (*α* = 0.92).

##### Cognitive control and flexibility

The CCFQ was used to measure cognitive control over emotion (*α* = 0.91) and appraisal and coping flexibility (*α* = 0.90) processes.

#### Statistical Analyses

ESEM was used to confirm the structure of the CCFQ. ESEM was used, as opposed to confirmatory factor analysis (CFA), because CFA has a strict requirement of zero cross-loadings in models with more than one substantive factor, which is an overly restrictive assumption that often leads to poor model fit. In ESEM, the assumption of zero-cross loadings is relaxed. That is, cross-loadings are freely estimated in the ESEM model akin to how cross-loadings are estimated in exploratory factor analysis. In addition, since the hypothesized two factors underlying the CCFQ are confounded by valence of item wording (the appraisal and coping flexibility factor has only positively worded items and the cognitive control over emotion consists mainly of negatively worded items), we included a method factor in the model to account for variance related to the valence of the items. This was accomplished by having all the negatively worded items load onto an additional third factor in the hypothesized model. For model identification purposes, the method factor was not allowed to correlate with the two substantive factors and the variance of the method factor was standardized (i.e., fixed to 1).

In terms of testing strategy, we examined whether the hypothesized two-factor model (that includes the method factor for the negatively worded items) provides a better fit to the data relative to a more parsimonious single-factor model (that includes a method factor for the negatively worded items). We also examined whether a more complex three-factor model (that also includes a method factor for the negatively worded items) provides a better fit to the data relative to the hypothesized two-factor model (that also includes the method factor for the negatively worded items). Mplus v.8.0 (Muthén and Muthén, 1998–2017) and robust maximum likelihood (MLR) estimation were used for all ESEM analyses to minimize the influence of non-normality on the estimation of the SEs in the model. The χ2 test of model fit, comparative fit index (CFI), and root mean square error of approximation (RMSEA) were used to adjudicate fit in the ESEM analyses. An excellent model fit would be reflected by a statistically non-significant χ2, a CFI close to 1, and RMSEA of.05 or less (see Kline, 2016). Models were compared using chi-square difference tests (Δχ2) with appropriate re-scaling.

Pearson correlation coefficients were used to examine the relationship between CCFQ subscale scores, stressor appraisals, coping and depressive symptoms. Multiple mediation analyses were conducted using the PROCESS v3.0 add-on to SPSS provided by Hayes (2017). Standardized predictor, mediator, and outcome variables were used in Model 4 of PROCESS, with 95% Confidence Intervals (*C.I*.) and 5000 bootstrap samples. Further, as males and females typically vary in the severity of depressive symptoms, all mediation analyses included participant gender as a covariate.

### Results and Discussion

#### Factor Structure of the CCFQ

The hypothesized model provided a marginal fit to the data, χ2 (112) = 223.693, *p* < 0.0001, CFI = 0.958, and RMSEA = 0.052. Modification indices suggested that including four correlations between the residual/error variances of several items would improve model fit. The residual correlations were between: (1) “*My thoughts and emotions interfere with my ability to concentrate*” and “*I get easily distracted by upsetting thoughts or feelings*,” (2) between “*I consider the situation from multiple viewpoints before responding*” and “*I take the time to see things from different perspectives before reacting*,” (3) between “*I can remain in control over my thoughts and emotions*” and “*I control my thoughts and feelings by putting the situation in context*,” and (4) between “*I take the time to think of several ways to best cope with the situation before acting*” and “*I control my thoughts and feelings by putting the situation in context.*” Because the wording for each pair of items was similar, we elected to include the residual correlations in the model to account for additional method variance.

Notably, the hypothesized model with the residual correlations provided a good fit to the data, χ2(108) = 132.353, *p* = 0.06, CFI = 0.991, and RMSEA = 0.025. Indeed, including the four residual correlations improved model fit, Δχ2(4) = 100.795, *p* < 0.001. The magnitude of the residual correlations was small-to-moderate and ranged from 0.242 to 0.493 in absolute value.

Next, we tested the fit of the alternative single-factor model and included the same four residual correlations. This model provided a poor fit to the data, χ2 (126) = 456.990, *p* < 0.0001, CFI = 0.875, and RMSEA = 0.084. As well, the alternative model provided a worse fit to the data relative to the hypothesized model with the four residual correlations, Δχ2(14) = 210365, *p* < 0.001. Likewise, the three-factor alternative model which included the residual correlations failed to converge to a unique solution due to a non-positive definite latent variable covariance matrix and thus fit statistics are unavailable. Nevertheless, this result suggests that three substantive factors do not underlie the CCFQ. In particular, it appears that the items comprising the CCFQ which assess appraisals and those assessing coping do not sufficiently (i.e., are not sensitive enough) distinguish between these processes, and thus converge on a single factor – *appraisals and coping flexibility*. In sum, the two-factor model with the four residual correlations provided the best absolute fit and relative fit to the data. Of particular significance is that the pattern of standardized factor loadings for each factor derived from the hypothesized model (see Table 5) replicates the loading pattern observed in Study 1. Also, as in Study 1, the two CFQ factors were moderately and positively correlated, *r* = 0.49, *p* < 0.001.

**Table 5:** Standardized factor loadings from the ESEM analysis of the CCFQ.

Item	Appraisal and coping flexibility	Cognitive control over emotion	Method
(1) I weigh out many options before choosing how to take action.	**0.621^∗∗^**	-0.056	—
(2) I control my thoughts and feelings by putting the situation in context.	**0.448^∗∗^**	0.206^∗∗^	—
(3) I take the time to see things from different perspectives before reacting.	**0.651^∗∗^**	0.025	—
(4) I consider the situation for multiple viewpoints before responding.	**0.706^∗∗^**	-0.053	—
(5) I can think of multiple coping options before deciding how to respond.	**0.637^∗∗^**	0.158†	—
(6) I take the time to think of more than one way to resolve the problem.	**0.751^∗∗^**	-0.015	—
(7) I manage my thoughts or feelings by reframing the situation.	**0.562^∗∗^**	0.267^∗∗^	—
(8) I take the time to think of several ways to best cope with the situation before acting.	**0.694^∗∗^**	0.208^∗∗^	
(9) I approach the situation from multiple angles.	**0.133†**	0.018	—
(10) I find it easy to set-aside unpleasant thought or emotions.	0.061	**0.672^∗∗^**	—
(11) It is easy for me to ignore distracting thoughts.	0.129	**0.599^∗∗^**	—
(12) I can remain in control over my thoughts and emotions.	0.174^∗^	**0.582^∗∗^**	—
(13) It’s difficult let go of intrusive thoughts or emotions.	-0.096	**0.644^∗∗^**	0.187^∗^
(14) I have a hard time managing my emotions.	0.046	**0.672^∗∗^**	0.529^∗∗^
(15) I feel like I lose control over my thoughts and emotions.	0.089	**0.638^∗∗^**	0.590^∗∗^
(16) It’s hard for me to shift my attention away from negative thoughts or feelings.	-0.037	**0.784^∗∗^**	0.299^∗∗^
(17) I get easily distracted by upsetting thoughts or feelings.	-0.041	**0.785^∗∗^**	0.176^∗^
(18) My thoughts and emotions interfere with my ability to concentrate.	-0.007	**0.735^∗∗^**	0.181^∗∗^


*Note: ESEM, exploratory structural equation modeling. ^†^*p* = 0.05; ^∗^*p* < 0.05; ^∗∗^*p* < 0.01, bold font indicates the factor in which the items were included.*

### Relationship Between the CCFQ, Stressor Appraisals, Coping Styles, and Depressive Symptoms

As shown in Table 6, individuals who reported greater levels of cognitive control and flexibility on the CCFQ tended to appraise a personally meaningful academic challenge more positively. Greater scores on both subscales of the CCFQ were related to less perceived threat; instead the situation was appraised as a challenge and more controllable. This was particularly evident among individuals who scored high on the cognitive control over emotion subscale of the CCFQ. Neither component of the CCFQ was related to centrality (i.e., the importance of the situation to the individual) or control-by-others. Furthermore, whereas problem-focused coping was more aligned with the appraisal and coping flexibility subscale, emotion-focused and avoidant coping were more strongly linked to the cognitive control over emotion subscale of the CCFQ. Together, these findings suggest that the CCFQ taps into processes related to effective coping.

**Table 6:** Relationship between the CCFQ, stressor appraisals, coping style, and symptoms of depression.

	1	2	3	4	5	6	7	8	9	10	11	12
(1) Cognitive control over emotion	–	–	–	–	–	–	–	–	–	–	–	–
(2) Appraisal and coping flexibility	0.52^∗∗∗^	–	–	–	–	–	–	–	–	–	–	–
(3) Threat	-0.36^∗∗∗^	-0.21^∗∗∗^	–	–	–	–	–	–	–	–	–	–
(4) Challenge	0.24^∗∗∗^	0.20^∗∗∗^	-0.25^∗∗∗^	–	–	–	–	–	–	–	–	–
(5) Centrality	0.09	-0.01	0.48^∗∗∗^	0.24^∗∗∗^	–	–	–	–	–	–	–	–
(6) Control-by-self	0.25^∗∗∗^	0.19^∗∗∗^	-0.46^∗∗∗^	0.54^∗∗∗^	0.04	–	–	–	–	–	–	–
(7) Control-by-others	0.01	0.03	-0.14^∗∗∗^	0.33^∗∗∗^	0.05	0.39^∗∗∗^	–	–	–	–	–	–
(8) Uncontrollable	-0.16^∗∗^	-0.04	0.45^∗∗∗^	-0.17^∗∗^	0.15^∗∗^	-0.47^∗∗∗^	-0.23^∗∗∗^	–	–	–	–	–
(9) Problem-focused coping	0.18^∗∗^	0.32^∗∗∗^	-0.19^∗∗∗^	0.34^∗∗∗^	-0.04	0.31^∗∗∗^	0.33^∗∗∗^	-0.14^∗∗^	–	–	–	–
(10) Emotion-focused coping	-0.61^∗∗∗^	-0.34^∗∗∗^	0.42^∗∗∗^	-0.18^∗∗^	0.16^∗∗^	-0.23^∗∗∗^	-0.07	0.29^∗∗∗^	-0.07	–	–	–
(11) Avoidant coping	-0.41^∗∗∗^	-0.11^∗^	0.23^∗∗∗^	-0.05	0.17^∗∗^	-0.06	0.04	0.17^∗∗∗^	0.25^∗∗∗^	0.54^∗∗∗^	–	–
(12) Depressive symptoms	-0.57^∗∗∗^	-0.39^∗∗∗^	0.43^∗∗∗^	-0.28^∗∗∗^	0.15^∗∗^	-0.37^∗∗∗^	-0.15^∗∗^	0.33^∗∗∗^	-0.30^∗∗∗^	0.63^∗∗∗^	0.33^∗∗∗^	–


*^∗^*p* < 0.05, ^∗∗^*p* < 0.01, ^∗∗∗^*p* < 0.001.*

It is important to mention that the CCFQ measures qualitatively distinct features to that of the SAM and SCOPE. The SAM and SCOPE assess the ‘content’ of an appraisal (e.g., levels of threat or control) or a coping strategy (i.e., frequency of endorsing emotional expression), respectively. By contrast, the CCFQ assesses the ‘processes’ of appraising, reappraising, and coping (i.e., generating multiple alternative appraisals and coping methods), independent of content (i.e., the use of a particular coping strategy). Thus, although all three measures assess aspects of ‘coping effectiveness,’ each measure taps into distinct factors.

Multiple mediation analyses were conducted to determine whether certain stressor appraisals and coping strategies mediated the relation between CCFQ scores and depressive symptoms. In the analyses concerning stressor appraisals, all 6 appraisal dimensions were entered simultaneously as proposed mediators, and for analyses involving coping, all 3 forms of coping were considered together. The total effect of cognitive control over emotion on depressive symptoms was *c* = -0.572, *SE* = 0.043, *p* < 0.001. Although cognitive control over emotion was associated with several appraisal dimensions, only uncontrollability uniquely mediated the relation between cognitive control over emotions and depressive symptoms (Table 7). The direct effect of cognitive control over emotion on depressive symptoms remained significant after accounting for all 6 appraisal dimensions, *c’* = -0.469, *SE* = 0.044, *p* < 0.001.

**Table 7:** Multiple mediations analyses examining the direct and indirect effects of CCFQ subscale scores on depressive symptoms through stressor appraisals and coping style.

		Depressive symptoms (*Y*)									
		
		*a*_i_ path			*b*_i_ path			Indirect effect (*a*_i_*b*_i_)			
				
Independent variable (*X*)	Mediator	Coefficient	*SE*	*p*	Coefficient	*SE*	*p*	Coefficient	Boot *SE*	Lower	Upper
Cognitive control over emotion	Threat	**-0.363**	**0.049**	**0.000**	0.099	0.059	0.097	-0.0352	0.0216	-0.0805	0.0061
	Challenge	**0.243**	**0.051**	**0.000**	-0.071	0.052	0.167	-0.0173	0.0131	-0.0441	0.0078
	Centrality	-0.088	0.052	0.090	0.060	0.051	0.247	-0.0040	0.0053	-0.0172	0.0043
	Control-by-self	**0.254**	**0.051**	**0.000**	-0.085	0.057	0.141	-0.0219	0.0191	-0.0663	0.0097
	Control-by-others	0.008	0.052	0.874	-0.046	0.045	0.308	-0.0015	0.0039	-0.0103	0.0060
	Uncontrollability	**0.159**	**0.051**	**0.002**	**0.137**	**0.049**	**0.006**	**-0.0202**	**0.0121**	**-0.0485**	**-0.0021**
Appraisal & coping flexibility	Threat	**-0.202**	**0.051**	**0.001**	**0.190**	**0.062**	**0.003**	**-0.0362**	**0.0153**	**-0.0701**	**-0.0104**
	Challenge	**0.202**	**0.051**	**0.001**	-0.097	0.055	0.078	-0.0192	0.0127	-0.0474	0.0027
	Centrality	0.001	0.052	0.984	0.054	0.055	0.320	0.0007	0.0043	-0.0077	0.0106
	Control-by-self	**0.190**	**0.052**	**0.003**	-0.091	0.062	0.141	-0.0169	0.0154	-0.0517	0.0098
	Control-by-others	0.025	0.052	0.638	-0.022	0.048	0.644	-0.0008	0.0033	-0.0081	0.0060
	Uncontrollability	0.051	0.052	0.329	**0.146**	**0.053**	**0.006**	-0.0060	0.0095	-0.0283	0.0113


*Note: Bold font indicates significant effects.*

The total effect of appraisal and coping flexibility on depressive symptoms was *c* = -0.389, *SE* = 0.048, *p* < 0.001. As shown in Table 7, although the appraisal and coping flexibility component of the CCFQ was related to multiple stressor appraisals, the relation between the appraisal and coping flexibility and depressive symptoms was uniquely mediated by threat appraisal. Yet, the direct effect of appraisal and coping flexibility on depressive symptoms remained significant after accounting for all 6 appraisal dimensions, *c’* = -0.303, *SE* = 0.045, *p* < 0.001. The present findings indicate that, although greater cognitive control and flexibility (as assessed by the CCFQ) was associated with multiple dimensions of stressor appraisals, only perceptions of threat and control were particularly important to depressive symptoms. These data are consistent with the view that these specific appraisal dimensions are particularly linked to depressive characteristics, and speak to the tendency to interpret (negative) life events as particularly threatening and beyond the individual’s control in promoting elevated and sustained depressive affect (Beck et al., 1979; Folkman and Lazarus, 1986; Abramson et al., 1989). Moreover, although the present findings are correlational, these results raise the possibility that lower levels of cognitive control and flexibility might be associated with a tendency to interpret stressful situations in a negative perspective. However, it might be the propensity for individuals who display difficulties of cognitive control and flexibility to specifically appraise stressor as threatening and uncontrollable that promotes sustained negative affect.

As displayed in Table 8, problem-focused and emotion-focused coping uniquely mediated the relation between greater cognitive control over emotion and lower depressive symptoms, although the direct effect of cognitive control of emotion on depressive symptoms remained significant after accounting for all 3 forms of coping, *c’* = -0.244, *SE* = 0.048, *p* < 0.001. Similarly, the relation between appraisal and coping flexibility and depressive symptoms was mediated by problem-focused and emotion-focused coping, but not avoidant coping (Table 8). Once again, the direct effect of appraisal and coping flexibility on depressive symptoms remained significant after accounting for all 3 forms of coping, *c’* = -0.124, *SE* = 0.043, *p* = 0.004.

**Table 8:** Multiple mediations analyses examining the direct and indirect effects of CCFQ subscale scores on depressive symptoms through coping style.

		Depressive Symptoms (*Y*)									
		
		*a*_i_ path			*b*_i_ path			Indirect Effect (*a*_i_*b*_i_)			
				
Independent variable (*X*)	Mediator	Coefficient	*SE*	*p*	Coefficient	*SE*	*p*	Coefficient	Boot *SE*	Lower	Upper
Cognitive control over emotion	Problem-focused	**0.175**	**0.052**	**0.001**	**-0.237**	**0.040**	**0.000**	**-0.0420**	**0.0170**	**-0.0797**	**-0.0124**
	Emotion-focused	**-0.606**	**0.042**	**0.000**	**0.439**	**0.051**	**0.000**	**-0.2623**	**0.0404**	**-0.3444**	**-0.1890**
	Avoidant coping	**-0.408**	**0.048**	**0.000**	0.045	0.048	0.340	-0.0181	0.0184	-0.0531	0.0195
Appraisal and coping flexibility	Problem-focused	**0.324**	**0.050**	**0.000**	**-0.243**	**0.042**	**0.000**	**-0.0786**	**0.0189**	**-0.1187**	**-0.0442**
	Emotion-focused	**-0.337**	**0.049**	**0.000**	**0.513**	**0.048**	**0.000**	**-0.1635**	**0.0325**	**-0.2287**	**-0.1001**
	Avoidant coping	**-0.110**	**0.052**	**0.035**	0.085	0.048	0.075	-0.0080	0.0066	-0.0232	0.0020


*Note: Bold indicates significant effects.*

The present findings suggest that greater cognitive control/flexibility might be associated with the endorsement of more effective coping strategies, and that the relationship between reduced cognitive control/flexibility (lower score on the CCFQ) and heightened depressive symptoms, might be partially accounted for by endorsement of ineffective coping methods. These findings are in line with reports that more frequent use of emotion-focused and avoidant coping, and limited engagement of problem-focused methods, were notable among depressed individuals (e.g., Whatley et al., 1998; Matheson and Anisman, 2003; Abdollahi et al., 2018). Once again, given the cross-sectional nature of the present data, these findings should not be interpreted as suggesting causality. Rather, these results indicate that a proportion of the relationship between CCFQ scores and depressive symptoms is accounted for by differences in stressor appraisals and the endorsement of particular coping methods.

## Study 3. Acute Stressor Effects on Cognitive, Affective, and Cortisol Responses: Moderating Role of the CCFQ

Reduced cognitive control and flexibility was associated with greater negative appraisals of stressful situations, which, in turn, was tied to more severe depressive symptoms (Study 2). Accordingly, it follows that when confronted with an acute stressor, individuals with lower levels of cognitive control/flexibility would appraise the challenge as more stressful and display more negative affect following the experience. To investigate this hypothesis, individuals with varying levels of cognitive control/flexibility based on the CCFQ were exposed to a psychosocial stressor (the Trier Social Stress Test; TSST) and were subsequently asked to appraise the challenge and report their current mood state.

Cognitive control processes, including cognitive reappraisal, not only play a central role in emotion regulation, but might also be associated with neuroendocrine and brain functioning (Ochsner et al., 2012; Compton et al., 2013; Denson et al., 2014; Silvers et al., 2015). In particular, it was surmised that prefrontal cortical top–down control contributes to the inhibition of responses generated by limbic functioning, which might include emotional responses and neuroendocrine outcomes. Thus, we determined whether individual differences in cognitive control/flexibility moderated the effects of an acute stressor on cortisol changes provoked by the TSST (Kirschbaum et al., 1993). The cortisol response elicited by acute stressors is thought to reflect an adaptive response to meet acute environmental or emotional demands (Sapolsky et al., 2000). Thus, it was hypothesized that higher CCFQ scores would be associated with a less pronounced cortisol changes in response to the TSST challenge. This study was restricted to females because of their elevated propensity to depression relative to that seen in males.

### Materials and Methods

#### Participants and Procedure

Participants comprised (*n* = 47) female undergraduate students, ranging in age from 18 to 26 years (*M*_age_ = 19.00, *SD*_age_ = 1.56). Self-reported ethnicities included Caucasian (660%, *n* = 31), South Asian (2.1%, *n* = 1), Arab/West Asian (6.4%, *n* = 3), South East Asian (2.1%, *n* = 1), Hispanic (2.1%, *n* = 1), Black (4.3%, *n* = 2), Asian (2.1%, *n* = 1), Aboriginal (2.1%, *n* = 1), and other (4.3%, *n* = 2). None of the participants reported a current physical illness/condition, nor were any of the participants taking anti-anxiety or antidepressant medications. Almost half of the participants (*n* = 20) were taking an oral contraceptive.

#### Laboratory Session

Laboratory sessions were conducted between 1300 and 1730 h to minimize the contribution of circadian factors to the cortisol responses. Participants were asked not to eat, drink (with the exception of water), or smoke for at least an hour before arriving to the session. Once signed informed consent was obtained, participants filled out several questionnaires concerning demographic information, general health, and medication history (e.g., antidepressants). Following the 30-min habituation period involved in completing these measures, participants provided a baseline saliva sample (for cortisol determination) and were randomly assigned to either the stressor or control condition. The control condition involved reading non-stressful magazines (e.g., O the Oprah magazine) for 15 min. Further saliva samples were collected at 5, 15, and 30 min following the stressor or control tasks.

#### The Trier Social Stress Test (TSST)

The TSST is a laboratory task designed to elicit psychological and physiological stress responses. Participants were told that they would engage in a public speaking task (about applying for a research assistantship), and given 5 min to prepare, after which they made their presentation in front of a panel of graduate student judges. Thereafter, participants engaged in an arithmetic task for 5 min. This consisted of participants being asked to subtract by 17, beginning with the number 1762. Participants were also told they were being videotaped during the TSST. Once the TSST (or control task) were completed, participants filled out several questionnaires concerning stressor appraisals, mood state, and cognitive flexibility.

#### Measures

##### Stressor appraisals

The SAM (Peacock and Wong, 1990) was used to assess perceived stressfulness of the task, where higher scores represent greater perceived stressfulness (*α* = 0.81).

##### Negative mood

The negative affect subscale of the Positive and Negative Affect Schedule-Expanded Version (PANAS-X; Watson and Clark, 1999) was used to assess the intensity of state negative mood immediately post-TSST. Responses ranged on a six-point scale from 0 (not at all) to 6 (extremely), with higher scores indicating greater intensity of negative affect (*α* = 0.89).

##### Cognitive control and flexibility

The 18-item CCFQ was used to measure individual differences in cognitive control over emotion (*α* = 0.88) and appraisal and coping flexibility (*α* = 0.91). The correlation between the two CCFQ components was *r* = 0.34, *p* < 0.05.

#### Salivary Cortisol

Saliva samples were frozen at -80°C until assayed for cortisol levels. Following the manufacturer’s protocol, a competitive radioimmunoassay, ^125^I kit (ICN Biomedicals Inc., Irvine, CA, United States), was used to determine, in duplicate, salivary cortisol levels. The intra- and inter-assay variability was <10%. The minimum detectable level of cortisol was 0.02 μg/dl and the specificity was 100%.

#### Statistical Analyses

Moderation analyses were conducted using the PROCESS v3.0 script to SPSS provided by Hayes (2017). Using Model 1 (to assess interactions), stressor condition was entered as the predictor variable, unstandardized CCFQ scores were entered as the moderating variable, and unstandardized appraisal, mood, and cortisol responses were entered as the outcome variables. Given the correlation between the components of the CCFQ, in the moderation analyses, when assessing the interactive effects of one component of the CCFQ (e.g., cognitive control over emotion), the second component (e.g., appraisal and coping flexibility) was treated as a covariate. Results without this procedure are presented in Supplementary Material (Supplementary Analyses: Study 3). Furthermore, as oral contraceptives can influence cortisol responses to a stressor (e.g., Kirschbaum et al., 1999; Mordecai et al., 2017), this variable was treated as a covariate in analyses concerning cortisol response.

Cortisol Area Under the Curve *increase* (AUC*i*; Pruessner et al., 2003) was computed to examine changes, represented by a single value, in cortisol levels elicited by the stressor and control tasks. In the formula below, *Cort* represents the absolute cortisol value in μg/dl and *T* refers to the length of time between cortisol sample collections. For example, *T1* represents the length of time between the collection of cortisol sample 1 (*Cort1*) and sample 2 (*Cort2*). *T1* = 20 min; *T2* = 10 min; *T3* = 15 min.

$$AUCi=(\frac{\left(Cort2+Cort1\right)}{2\times T1}+\frac{\left(Cort2+Cort1\right)}{2\times T2}+\frac{\left(Cort2+Cort1\right)}{2\times T3})-\left(Cort1\times \left(T1+T2+T3\right)\right)$$

### Results and Discussion

The TSST challenge was appraised as being more stressful (main effect of condition: *b* = 3.16, *SE* = 0.93, *p* = 0.001) relative to reading a magazine in the control condition. However, contrary to expectations, individual differences in the appraisal and coping flexibility, Δ*R*^2^ = 0.03, *F*(1,42) = 3.07, *p* = 0.09, and cognitive control over emotion, Δ*R*^2^ = 0.00, *F*(1,42) = 0.16, *p* = 0.69, components of the CCFQ did not moderate this effect. Thus, cognitive flexibility as assessed by the CCFQ might not contribute to differences in overall perceived stressfulness concerning an acute challenge. Perceived stressfulness, however, was related to greater negative affect (*r* = 0.74, *p* < 0.001) and, although not quite statistically significant, elevated cortisol AUCi (*r* = 0.30, *p* = 0.06).

**FIGURE 1:**
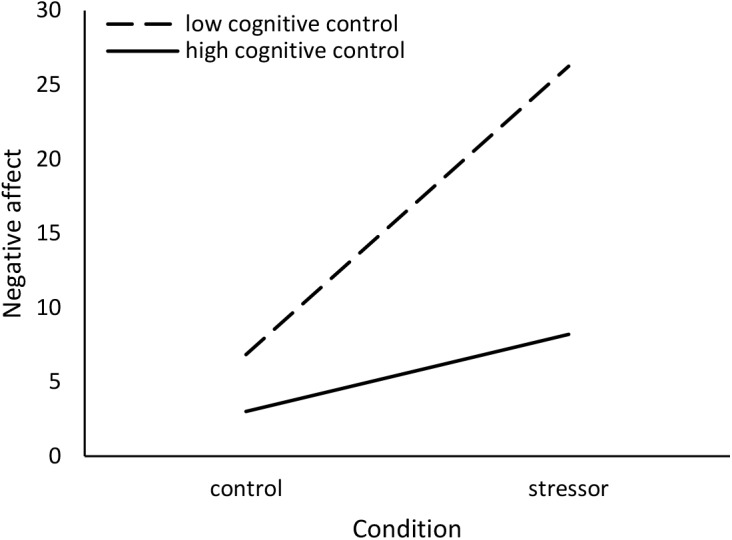
Moderating effect of cognitive control over emotion in the relation between stressor condition and negative affect. Low cognitive control over emotion = 1 *SD* below the mean, high cognitive control over emotion = 1 *SD* above the mean.

As predicted, the TSST elicited greater negative affect than the control condition (*b* = 37.17, *SE* = 9.43, *p* < 0.001), but this effect depended on differences of cognitive control over emotion, Δ*R*^2^ = 0.08, *F*(1,42) = 0.7.35, *p* < 0.01. As shown in Figure 1, among individuals reporting low cognitive control over emotion, the TSST elicited greater negative affect compared to the control condition (*b* = 19.40, *SE* = 3.41, *p* < 0.001). By contrast, among individuals with high cognitive control over emotion, the TSST and control condition provoked equally low levels of negative affect (*b* = 5.18, *SE* = 3.40, *p* = 0.14). These findings were unique to cognitive control over emotion, as the appraisal and coping flexibility component of the CCFQ did not moderate the effects of stressor condition on negative affect, Δ*R*^2^ = 0.02, *F*(1,42) = 1.72, *p* = 0.20. Thus, in line with the findings presented in Study 2, when confronted with a stressful situation, greater ability to disengage (or shift attention away) from negative thoughts and emotions (as measured by the cognitive control over emotion dimension of the CCFQ) might be accompanied by less intense negative mood or increased emotional regulation.

**FIGURE 2:**
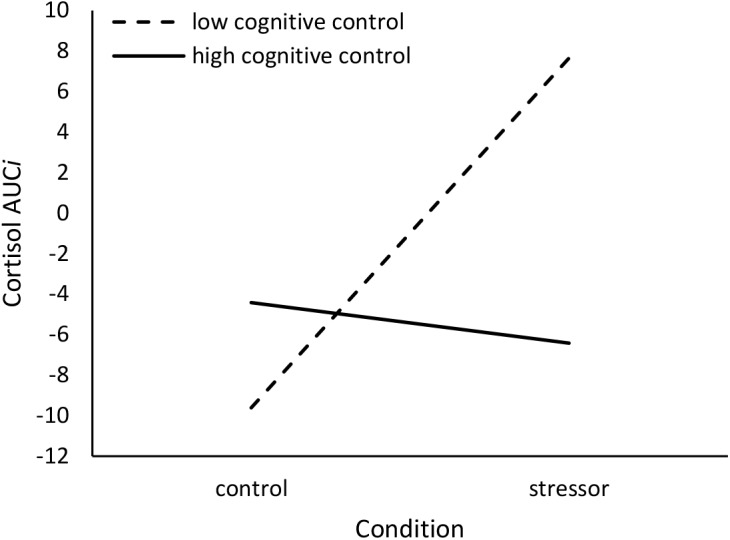
Moderating effect of cognitive control over emotion in the relation between stressor condition and cortisol Area Under the Curve increase (AUC*i*). Cortisol AUC*i* represents the relative change in cortisol levels (in μg/dl) from baseline. Low cognitive control over emotion = 1 *SD* below the mean, high cognitive control over emotion = 1 *SD* above the mean.

The Stressor Condition × Cognitive Control over Emotion interaction accounted for considerable variance in cortisol response, Δ*R*^2^ = 0.08, *F*(1,35) = 3.67, *p* = 0.06. As shown in Figure 2, in comparison to the control condition, the TSST elicited greater cortisol levels (i.e., larger cortisol AUC*i* index) among individuals with low cognitive control over emotion (*b* = 17.25, *SE* = 6.84, *p* = 0.02), but not among those with high cognitive control over emotion (*b* = -1.99, *SE* = 6.81, *p* = 0.77). This effect was unique to cognitive control over emotion as the variation in appraisal and coping flexibility did not moderate the effects of stressor condition on cortisol response, Δ*R*^2^ = 0.01, *F*(1,35) = 0.24, *p* = 0.63. Moreover, when not controlling for the appraisal and coping flexibility, the Stressor Condition × Cognitive Control over Emotion interaction was slightly weaker, Δ*R*^2^ = 0.06, *F*(1,36) = 2.56, *p* = 0.12. Thus, greater cognitive control over emotion, or the ability to disengage from negative cognitive and emotional states, might not only serve to regulate emotions effectively, but might also play a role in regulating the cortisol response associated with a stressful situation.

It is not entirely clear why the cognitive control over emotion, but not the appraisal and coping flexibility component of the CCFQ, uniquely moderated the cortisol response to the TSST. However, these findings are consistent with previous reports indicating that trait rumination, which has been associated with diminished cognitive control over emotion (Koster et al., 2011), predicted delayed cortisol recovery following an acute psychosocial stressor (Zoccola et al., 2010; Stewart et al., 2013). In contrast, cognitive reappraisal, potentially because of the degree of effort required to engage in this emotion regulation strategy, was associated with elevated cortisol reactivity (Denson et al., 2014). Indeed, these findings might also partly explain why the moderating effect of the cognitive control over emotion component of the CCFQ was stronger when the appraisals and coping component was controlled for.

Because the present version of CCFQ assesses “trait” cognitive control/flexibility, it was not possible to determine whether individuals actually engaged in these behaviors during the stressor session, although trait measures have been reported to predict responses to acute stressors (e.g., Zoccola et al., 2010; Stewart et al., 2013). This aside, the main purpose of the present study was to determine whether the CCFQ, as a measure of cognitive control and flexibility in stressful situations, would effectively predict cognitive, emotional, and physiological responses to an acute stressor, which was, in fact, found to occur. However, as the present study included only females, it is remains uncertain whether a similar pattern of results would be observed amongst males.

## General Discussion

The present research examined how cognitive control and cognitive flexibility might be expressed in stressful situations and the processes through which reductions in these abilities might be linked to elevated depressive symptoms. To this end, the CCFQ was developed was developed to assess an individual’s perceived levels of cognitive control and flexibility that were displayed through three stress-related processes, including attention, appraisals and reappraisals, and coping.

The CCFQ comprised two distinct, yet overlapping dimensions of cognitive control and flexibility, which exhibited good internal reliability and construct validity. The *cognitive control over emotion* dimension of the CCFQ assessed the extent to which an individual perceives that they can control intrusive and repetitive (primarily negative) thoughts and emotions that are ordinarily elicited by a stressful situation. As assessed by this dimension of the CCFQ, individuals who perceive having greater cognitive control over emotion more readily shift their attention away from negative cognitive and emotional states, allowing them to focus their efforts on directly resolving a stressful situation. In contrast, individuals with perceived low cognitive control over emotion are more likely to engage in repetitive negative thinking and the excessive processing of negative emotions. As assessed by the CCFQ, reduced cognitive control over emotion was associated with increased repetitive thinking and rumination, elevated negative affect following a stressful situation, and ultimately heightened symptoms of depression. Furthermore, diminished cognitive control over emotion was linked to elevated and prolonged cortisol reactivity following an acute challenge, a neuroendocrine profile that has been associated with depressive illness (Juruena et al., 2017).

The *appraisal and coping flexibility* dimension of the CCFQ assessed an individual’s perceived ability to engage in a set of deliberate effortful behaviors that can facilitate a comprehensive and favorable appraisal of a stressful situation as well as the selection of a broad range of coping strategies. In particular, greater scores on the appraisal and coping flexibility dimension represent a tendency to approach stressful situations from multiple perspectives prior to responding, manage negative thoughts and emotions by reframing or reappraising stressful situations, and generate multiple and alternative coping strategies prior to selecting the appropriate response. Low appraisal and coping flexibility, by contrast, reflects a tendency to respond too readily or automatically, resulting in more reactive cognitive, emotional, and behavioral responses. In the present study, diminished appraisal and coping flexibility were accompanied by negative stressor appraisals, lower reappraisal in the context of emotion regulation, the endorsement of ineffective and inflexible coping, and heightened depressive symptoms.

A central aim in developing the CCFQ was to examine aspects of cognitive control and of cognitive flexibility relevant to stressful situations that have yet to be explored using other measures, including the Cognitive Flexibility Inventory. It was expected that the two questionnaires would display some convergence given that they both assess aspects of cognitive flexibility. This said, the way in which the CCFQ and Cognitive Flexibility Inventory conceptualized and assessed cognitive flexibility differed, and thus these measures might be predictive of different processes. Specifically, the CCFQ explicitly and directly focused on the (negative) cognitive and emotional states elicited by a stressful situation, and the cognitive control processes required to regulate these responses. In turn, as indicated in Study 1, higher scores on the CCFQ were most strongly related to greater cognitive reappraisal as well as less perseverative thinking and rumination. In contrast, the Cognitive Flexibility Inventory focuses on challenging and replacing maladaptive thoughts with more balanced and adaptive thinking, and was more strongly related to coping flexibility and expressive suppression (emotional containment). Thus, although each measure might be useful in different settings, their concurrent use might be equally valuable in distinguishing different aspects of cognitive flexibility relevant to stress-related psychopathology.

Through the development of the CCFQ, the present research provided insights into how cognitive control and cognitive flexibility might be manifested in stressful situations, and whether reductions in these abilities might be accompanied by elevated symptoms of depression. To be sure, the CCFQ was not developed as a diagnostic instrument, but instead was intended to compliment behavioral paradigms in determining common as well as different aspects of cognitive control and flexibility that are disturbed among individuals with depressive pathology. From this perspective, distinguishing components of cognitive control as well as cognitive flexibility through both behavioral tasks and self-report measures, might offer clues regarding effective clinical treatment approaches (e.g., personalized/precision treatment) for depressive pathologies. Moreover, although the present research focused on depressive symptoms, the CCFQ might also be useful in relation to other psychiatric disorders that have been associated with impaired cognitive control and flexibility, including anxiety (e.g., obsessive–compulsive disorder), substance use, bipolar, and eating disorders (Cunha et al., 2010; Gruner and Pittenger, 2017; O’Donnell et al., 2017; Park and Moghaddam, 2017; Perpiñá et al., 2017).

## Ethics Statement

This study was carried out in accordance with the recommendations of Carleton University Ethics Committee for Psychological Research. The protocol was approved by the Carleton University Ethics Committee for Psychological Research. All subjects gave written informed consent in accordance with the Declaration of Helsinki.

## Author Contributions

RG was solely responsible for data acquisition. RG and NT were involved in data analysis. RG, KM, and HA contributed to the conception and study design, and all authors contributed to the interpretation, drafting, revising, and final approval of the present manuscript.

## Conflict of Interest Statement

The authors declare that the research was conducted in the absence of any commercial or financial relationships that could be construed as a potential conflict of interest.
