# Supplementary material for: Cognitive Control and Flexibility in the Context of Stress and Depressive Symptoms: The Cognitive Control and Flexibility Questionnaire
Source: Front Psychol. 2018 Nov 19;9:2219. doi: 10.3389/fpsyg.2018.02219 (PMC6252356; doi:10.3389/fpsyg.2018.02219)
Supplement: Supplementary file 1 [file Table_1.docx]

Supplementary Material

Cognitive Control and Flexibility in the context of Stress and Depressive Symptoms:

The Cognitive Control and Flexibility Questionnaire

Robert L Gabrys*, Nasim Tabri, Hymie Anisman, & Kimberly Matheson

*** Correspondence:** Robert Gabrys, Department of Neuroscience,

Carleton University, 1125 Colonel By Drive, Ottawa, Ontario, K1S 5B6, Canada.

Tel: (613) 520-2600 ext. 2692, email: [robert.gabrys@carleton.ca](mailto:robert.gabrys@carleton.ca)

**1 Supplementary Table 1**

Promax rotated, Principal Components Analysis of the initial 44 items of the CFQ-S

|  | Factor | | | | | | |
| --- | --- | --- | --- | --- | --- | --- | --- |
| Item | 1 | 2 | 3 | 4 | 5 | 6 | 7 |
| 1. **I take the time to think of more than one way to resolve the problem. (29)** | .83 | .29 | .48 | .09 | .17 | .16 | .10 |
| 1. I play out the consequences of various actions before choosing the best one. (25) | .81 | .38 | .43 | .16 | .21 | .13 | .09 |
| 1. I think of a plan of what to do best before acting. (19) | .79 | .35 | .30 | .03 | .19 | .11 | .03 |
| 1. **I approach the situation from multiple angles. (27)** | .79 | .23 | .47 | .11 | .14 | .22 | .16 |
| 1. **I consider the situation for multiple viewpoints before responding. (13)** | .76 | .21 | .46 | .13 | .01 | .24 | .33 |
| 1. I look for many strategies of dealing with the situation. (23) | .73 | .42 | .49 | .20 | .29 | .34 | .16 |
| 1. I play out the situation in my mind before responding. (32) | .73 | .39 | .37 | .03 | .37 | .10 | .05 |
| 1. **I take the time to think of several ways to best cope with the situation before acting. (44)** | .73 | .37 | .59 | .27 | .30 | .23 | .08 |
| 1. I anticipate the consequences of my actions before acting. (5) | .67 | .39 | .19 | -.14 | -.02 | -.08 | .25 |
| 1. **I manage my thoughts or feelings by reframing the situation. (33)** | .67 | .46 | .71 | .29 | .40 | .18 | .26 |
| 1. **I weigh out my options before choosing how to take action. (1)** | .65 | .38 | .33 | -.09 | .04 | .22 | .13 |
| 1. **I take the time to see things from different perspectives before reacting. (11)** | .64 | .25 | .47 | .15 | -.04 | .31 | .38 |
| 1. **I can easily think of multiple coping options before deciding how to respond. (22)** | .60 | .44 | .62 | .30 | .51 | .26 | .14 |
| 1. **I control my thoughts and feelings by putting the situation into context. (4)** | .55 | .58 | .53 | .07 | .11 | .14 | .34 |
| 1. I get rid of negative emotions by changing the way I think about the situation.* (24) | .55 | .41 | .74 | .23 | .30 | .15 | .31 |
| 1. I get rid of negative thinking by re-evaluating the situation. (18) | .52 | .39 | .76 | .21 | .27 | .08 | .23 |
| 1. I control negative thoughts and emotions by modifying the way I think about the situation. (17) | .47 | .46 | .79 | .25 | .28 | .07 | .27 |
| 1. I find it difficult to think of many options for resolving the situation.* (34) | .46 | .56 | .36 | .27 | .28 | .65 | -.04 |
| 1. **I can remain in control of my thoughts and emotions. (6)** | .44 | .74 | .45 | .26 | .38 | .14 | .33 |
| 1. I can easily deal with my thoughts and feelings. (31) | .39 | .73 | .56 | .39 | .56 | .22 | .15 |
| 1. It is easy for me to reassess a negative experience into a positive one. (42) | .39 | .43 | .79 | .45 | .51 | .53 | .10 |
| 1. **I feel like I lose control over my thoughts and emotions.* (14)** | .38 | .84 | .47 | .42 | .42 | .41 | .16 |
| 1. Putting a positive spin on a bad experience comes fairly easy to me. (38) | .36 | .41 | .81 | .39 | .43 | .41 | .12 |
| 1. It’s hard to think of different ways of dealing with the situation.* (3) | .35 | .55 | .35 | .26 | .33 | .69 | .24 |
| 1. I have difficulty controlling my thoughts and emotions.* (39) | .33 | .83 | .51 | .41 | .51 | .42 | .10 |
| 1. **I find it easy to set-aside unpleasant thought or emotions. (41)** | .33 | .53 | .65 | .47 | .70 | .35 | .06 |
| 1. I find it easy to look for something positive, even when I am stressed. (16) | .32 | .49 | .70 | .30 | .38 | .29 | -.05 |
| 1. **I have a hard time managing my emotions.* (10)** | .31 | .85 | .45 | .38 | .48 | .38 | .25 |
| 1. My thoughts and emotions become too much to deal with.* (9) | .29 | .83 | .44 | .43 | .40 | .40 | .30 |
| 1. I can’t think about anything else except for the situation.* (40) | .29 | .69 | .51 | .39 | .55 | .41 | -.12 |
| 1. It’s hard for me to put things in perspective when I’m upset.* (8) | .28 | .64 | .36 | .30 | .36 | .37 | .60 |
| 1. **It is easy for me to ignore distracting thoughts. (20)** | .26 | .49 | .42 | .37 | .78 | .34 | .10 |
| 1. I can’t stop dwelling on my feelings.* (36) | .23 | .70 | .50 | .70 | .60 | .39 | -.00 |
| 1. I can easily to suppress upsetting memories. (43) | .23 | .38 | .45 | .46 | .64 | .23 | .14 |
| 1. **It’s hard for me to shift my attention away from negative thoughts or feelings.* (15)** | .22 | .68 | .65 | .51 | .54 | .55 | .17 |
| 1. **My thoughts and emotions interfere with my ability to concentrate. * (28)** | .21 | .69 | .37 | .44 | .69 | .41 | .03 |
| 1. **I get easily distracted by upsetting thoughts or feelings.* (26)** | .20 | .71 | .45 | .50 | .66 | .52 | .24 |
| 1. It is easy for me to shift my attention to other things if I am upset. (30) | .18 | .52 | .45 | .23 | .59 | .15 | .27 |
| 1. I can’t focus on anything when I am upset.* (2) | .16 | .45 | .16 | .19 | .51 | .52 | .36 |
| 1. It’s hard for me to ignore negative emotions once they have been provoked.* (21) | .16 | .48 | .43 | .41 | .49 | .60 | .25 |
| 1. **It’s difficult let go of intrusive thoughts or emotions.* (7)** | .14 | .42 | .43 | .44 | .49 | .29 | .59 |
| 1. The same thoughts keep going through my mind again and again.* (35) | .13 | .45 | .39 | .87 | .41 | .27 | .12 |
| 1. My thoughts repeat themselves over and over again.* (37) | .08 | .49 | .39 | .89 | .45 | .35 | .09 |
| 1. I keep playing the situation over and over again in my head.* (12) | .03 | .28 | .28 | .77 | .38 | .31 | .11 |
| *Eigenvalue* | 16.73 | 4.94 | 2.07 | 1.47 | 1.21 | 1.11 | 1.03 |
| *Variance explained (%)* | 38.02 | 11.23 | 4.71 | 3.35 | 2.75 | 2.53 | 2.34 |

Note: Bold represents the final 18 items comprising the CCFQ; * indicates reverse coded items; number in parentheses represents the order in which the initial 44 items were administered.

**2** **Supplementary Analyses: Study 3**

In Study 3, we presented data examining the moderating effects of each component of the CCFQ on the effects of an acute stressor on several outcomes (i.e., appraisals, mood, and cortisol). In these analyses, when examining the interactive effects of one component of the CCFQ (e.g., cognitive control over emotion), the second component (e.g., appraisal and coping flexibility) was treated as a covariate. Here, we present the results *without* controlling for either component of the CCFQ.

*Stressfulness*

Similar to the findings presented in the manuscript, the Stressor x Cognitive Control interaction on Stressfulness was not significant, *ΔR^2^* = .00, *F*(1, 43) = .30, *p* = .59, and the Stressor x Appraisal/Coping Flexibility interaction on Stressfulness approached statistical significance, *ΔR^2^* = .03, *F*(1, 43) = .30, *p* = .07.

*Negative Affect*

The Stressor x Cognitive Control interaction on negative affect was significant, *ΔR^2^* = .08, *F*(1, 43) = 7.48, *p* < .001, and nearly identical to the results presented in the manuscript. Likewise, the Stressor x Appraisal/Coping Flexibility interaction on negative affect was not significant, ΔR^2^ = .03, *F*(1, 43) = 2.30, *p* = .14, paralleling the findings presented in the manuscript.

*Cortisol*

The Stressor x Cognitive Control interaction contributed to slightly less variance in cortisol AUCi, when not controlling for the Appraisal and Coping component of the CCFQ, *ΔR^2^* = .06, *F*(1, 36) = 2.56, *p* = .12. Once again, the Stressor x Appraisal/Coping Flexibility interaction on cortisol AUCi was not significant, *ΔR^2^* = .01, *F*(1, 34) = .35, *p* = .56.
